# The Efficacy of the “Dat-e Adolescence” Prevention Program in the Reduction of Dating Violence and Bullying

**DOI:** 10.3390/ijerph16030408

**Published:** 2019-01-31

**Authors:** Noelia Muñoz-Fernández, Javier Ortega-Rivera, Annalaura Nocentini, Ersilia Menesini, Virginia Sánchez-Jiménez

**Affiliations:** 1 Universidad Loyola Andalucía, 41014 Seville, Spain; nmunoz2@us.es; 2 Department of Developmental and Educational Psychology, University of Sevilla, 41807 Seville, Spain; javortega@us.es; 3 Department of Education, Languages, Intercultures, Literatures and Psychology, University of Florence, 50135 Florence, Italy; annalaura.nocentini@unifi.it (A.N.); ersilia.menesini@unifi.it (E.M.)

**Keywords:** dating violence, bullying, prevention program, Dat-e Adolescence

## Abstract

*Background*: The aim of this study was to assess the efficacy of the school-based “Dat-e Adolescence” prevention program in the reduction of dating aggression and victimization and bullying in adolescents. *Method*: a RCT design with three waves (pre-test, post-test and follow-up six months apart) and two groups (an experimental group and a control group) were used. One thousand four hundred and twenty three (1423) adolescents, mean age 14.98 (557 in the experimental group) participated in the study. *Results*: Efficacy evaluation was analyzed using Multiple-group latent growth models and showed that the Dat-e Adolescence program was effective in reducing sexual and severe physical dating violence and bullying victimization. *Conclusions*: The results suggest that dating violence prevention programs could be an effective approach for tackling different behavioral problems in adolescence given the protective and risk factors shared between dating violence and bullying.

## 1. Introduction

Dating violence is considered a subtype of intimate partner violence, which occurs in relationships that are more or less stable or lasting and encompasses aggressive behavior, be it psychological, physical, sexual, or via new technologies [1]. Although prevalence rates continue to be inconsistent, international studies have shown that psychological violence is the most frequent type, with percentages ranging from 20% to 80% [2,3], followed by physical forms at around 20%, and sexual violence at around 9% [4]. More recently, measures of online dating violence have yielded rates that vary from 2% to 50%, depending on the severity of the behavior under analysis [5]. Its consequences for adolescent development and health have also been highlighted, such as heavy episodic drinking, depressive symptomatology, suicidal ideation, smoking, and engaging in other antisocial behaviors [6]. Moreover, being involved in teen dating violence is associated with other behavioral problems such as bullying and sexual harassment. In this respect, studies have shown that bullying is a strong predictor of sexual harassment and dating violence in adolescence [7,8,9,10]. This association between dating violence and other forms of aggression has had various explanations. First, similar risk profiles for bullying and dating violence have been observed, such as attitudes towards violence and substance consumption [9]. Second, it has been hypothesized that the lessons learned in the peer relationship domain would be transferred to new contexts, such as romantic relationships [9,10], contributing to the internalization of an aggressive relational pattern [8,9,10]. In short, the prevalence and consequences of dating violence and its link to other behavioral problems have led us to define this phenomenon as a public health problem [1], which calls for preventive intervention to address these behaviors. Recognizing and considering its association with other, different behavioral problems would help to design ecological prevention programs that could improve their impact on adolescents’ social networks [1]. 

In recent decades there has been a growing number of evidence-based dating violence prevention programs, of which we can mention the Safe Dates program [11]; the Fourth-R [12] and Shifting Boundaries [13], among others. Recent systematic reviews and meta-analyses [14,15,16,17] have concluded that dating violence prevention programs are promising for changing attitudes toward violence and for increasing adolescents’ knowledge about the problem. However, the efficacy of these programs for reducing aggressive behavior has received less support and the results remain inconclusive. Research that has focused on synthetizing the available published literature has drawn two main conclusions: (1) few programs have shown positive effects in reducing dating aggression and victimization; and (2) not all forms of dating violence are sensitive to the treatment effects at the same level. In particular, three programs have reported changes in physical and/or sexual violence [13,18,19], whereas only one program has reported changes in psychological violence, namely the Safe Dates program [11,20]. Lastly, three studies [21,22,23] have identified changes in composite measures of different forms of abuse where psychological, physical and/or sexual violence were covered within the same measure. However, the absence of measure specificity makes it difficult to determine which outcome was responsible for the program’s efficacy. In general terms, research suggests that the majority of programs appear to reduce severe and less frequent forms of violence, such as physical and sexual violence. In contrast, the results are less consistent when it comes to more frequent forms of violence, as is the case of psychological violence.

Some other available programs have tried to prevent aggressive behavior in adolescence in a more general way, attending to the reduction of bullying, dating violence or other forms of violence such as sexual harassment [12,13,24]. These studies acknowledge that dating violence and bullying share protective and risk factors, such as attitudes toward violence, feelings of anger, poor communication skills, and high levels of conflict [25]. The intervention on these factors could impact on these different aggressive behaviors at the same time. From this perspective, these programs may be considered cross-cutting in nature because a single program endeavors to prevent multiple problem behaviors, promoting the skills and strategies that young people need to tackle different forms of violence. As for their efficacy, some of these programs have failed to observe a significant reduction in dating violence and bullying [24]; others have observed decreased physical dating aggression but no decrease in peer aggression [12]; and others have reported a reduction in sexual dating violence, sexual harassment, and peer sexual violence [13]. Although these programs are promising from a cost-benefit analysis standpoint, the controversial results obtained thus far prevent us from concluding on the impact that dating violence prevention programs could have on other forms of interpersonal violence. 

Considering the high variability found in terms of the efficacy of dating violence prevention programs, reviews and meta-analyses have identified some key aspects of the intervention design that appear able to be translated with greater success. Intervening during early adolescence (around age 13), when romantic relationships begin to show their importance in young people’s lives and violent behavior, for the most part, has not yet surfaced, is recommended [26]. Other authors see the benefits of starting even earlier, intervening on predictors of dating violence such as bullying and sexual harassment [9,10]. Research also suggests the need to act upon the mediating variables to change behaviors—that is, on those variables that longitudinal studies have identified as precursors of dating violence [26]. In contrast, other authors [27] have concluded that violence prevention in young couples should not only focus on specific risk factors but also adopt a positive framework to promote the development of social and emotional skills. Intervention duration is also another relevant component. Short, 90-minute, single-session programs showed moderate results compared to longer programs whose effects were more robust over time [28,29]. It is also crucial to design comprehensive interventions of high methodological quality and rigor. Interventions applied to more than one context (e.g., school, family, teaching, and community settings), which incorporate randomized controlled trials (hereafter RCT) reported greater efficacy than other evaluation trials [15,28]. 

In Europe and specifically in Spain, there are no dating violence prevention programs that have been developed with high methodological quality using random assignment methods and whose design and procedure allow us to verify their efficacy in a reliable way [2]. This scenario is surprising when compared to the evidence-based programs available in Europe and Spain to address the prevention of school bullying and cyberbullying, as is the case of KiVa in Finland [30]; NoTrap! in Italy [31] and ConRed in Spain [32], among others. Specifically, Spanish dating violence prevention programs have shown low methodological quality characterized by participant selection through intentional sampling [33] and the use of quasi-experimental designs [34,35,36]. Most of these programs included a very small number of participants which makes it difficult to draw generalizable conclusions [33,35,36]. Considerable variability was also found in relation to efficacy analysis; while some programs intervened and assessed their effectiveness in attitudinal changes, beliefs and knowledge, others focused on behavioral changes. Specifically, those programs whose objective was to modify attitudes, beliefs and knowledge toward violence were effective [33,34,35]. On the other hand, only two programs have evaluated the program’s effectiveness in relation to dating violence outcomes [35,36]. DaViPop [36] has been the only one to find positive results in the reduction of dating violence. This program also intervenes in romantic relationship quality, yielding positive results for support and future expectations. Within this framework, Dat-e Adolescence [37] has emerged as a response to the need for developing programs with high methodological quality and inspired by an evidence-based approach in the area of dating violence prevention in Spain. The aim of the present study was to examine the efficacy of the Dat-e Adolescence program (First Edition) in relation to reducing dating violence and bullying at follow-up measurement, that is, in the longer term as compared to the intervention implementation. 

### 1.1. Theoretical Background of Dat-e Adolescence 

Dating violence boasts specific characteristics that need to be taken into account. It is primarily mutual and bidirectional and related to conflicts in the dyad [17,38,39]. Studies have shown that this aggressive behavior is more common and less severe than other forms of violence in romantic relationships in adolescence, as gender-violence is, but it is clearly related [17]. At this respect, some of the risk factors that explain dating violence has an important role in gender- based violence. This is the case of peer context, which is not only crucial to the onset and maintenance of first romantic relationships [40], but also to violence in romantic relationships. Bullying and sexual harassment have been described as a stepping-stone to dating violence [9,10,41,42]. It has been observed that young people exposed to peer group violence tend to internalize acceptance of violence norms and develop a maladjusted perception of the consequences of violence on the victims, which poses a greater risk to violent behavior involvement in other relational contexts, including romantic relationships [43]. 

These characteristics are key aspects of the Dynamic Developmental Systems Model [44]. From a developmental and ecological perspective, this model acknowledges that dating violence is a complex problem associated with multiple risk factors [17]. It integrates different risk factors that contribute to violence among young couples (for a review of risk/protective factors, see [45,46]). Within this framework, abusive practices in the romantic relationship are seen as a consequence of the interaction that takes place among biological variables (e.g., genetic influences); individual variables (e.g., antisocial behavior); contextual factors (e.g., parental divorce); and the behaviors, beliefs and attitudes that each partner has acquired from socialization in different contexts (e.g., family, peers) throughout their life. Furthermore, in order to understand violence from this perspective, it is important to address not only the risk factors corresponding to each member of the couple, but also the relationship they develop and maintain; that is, their relational interactions and characteristics. Accordingly, violence is seen as a consequence of jealousy, gender beliefs and attitudes about violence; of an insecure and/or avoidant attachment with the partner; of low levels of support and intimacy in the relationship; and lack of skills and strategies for tackling the misunderstandings and conflicts that often arise in the couple. Because of this dynamic, violence becomes stable within the romantic relationship, since abusive practices are consolidated as relational forms within couples [17,47]. 

This main risk factors addressed by DDS Model are considered in Dat-e Adolescence program. From our point of view, if boys and girls reflect about violence, gender norms, how the peer network influences their behavior, and they develop socio-emotional skills to be confident within their romantic relationships, they will be capable to build healthy relationships free of dating violence and gender-based violence. This comprehensive approach seems to be promising for prevention programs when working at universal level in community samples because permits to deep on the different expressions of violence that can occur within the couple, their nature, causes and how to deal with it [12,26].

### 1.2. The Dat-e Adolescence Program

Dat-e Adolescence is a school-based universal and multi-component prevention program designed for adolescents aged 12 to 19 years. It comprises seven 1-hour long sessions that can be implemented during school hours. In general terms, the program adopts a constructivist and experiential approach that encourages content learning through different teaching and learning experiences. It includes researcher- and peer-led training:

*Researcher-led training.* The first five sessions are administered by researchers during school hours. The main aims of these lessons are to (a) raise awareness of the concepts of love, myths about romantic love, and healthy behaviors in relationships; (b) encourage greater recognition, expression, and emotional regulation; and (c) promote enhanced self-esteem. In terms of couple dynamics, the program aims to (d) improve communication skills; and (e) promote the recognition of violent behavior in both traditional and online forms of violence. These lessons comprise discussions, role-playing, debates, and watching videos to promote socio-emotional skills and increase knowledge about romantic relationships and abuse. They consist of classroom and web-based activities, the latter via the program’s online platform. 

*Peer-led training.* The last two sessions are administered by peers during school hours. In the fifth session, two students from each class (one boy and one girl) volunteer to be the implementers of these final two sessions. These assistant students received four hours’ training (two for each session) prior to the peer sessions. Presenting a conflictive or abusive context, these peer-led sessions aim to (a) raise awareness and promote coping strategies when aggression occurs and conflict-resolution strategies; and (b) raise awareness of the peer group and bystander’s influence in the face of dating violence. These lessons comprise decision-making games and group dynamic exercises. 

*School session.* A final activity is organized by the participating schools covering the main contents and lessons learned during the intervention. 

The efficacy of Dat-e Adolescence has been assessed over two large RCT waves [37]. The program significantly modified beliefs towards violence, specifically on myths about romantic love (Cohen’s *d* from −0.56 to −0.94); enhanced self-esteem (*d* = −0.15); and emotion regulation (*d* = −0.19) among participants. However, no significant effects were found for dating violence at post-test. 

The present study tests the program’s medium-term effect on moderate physical dating violence, severe physical dating violence and sexual dating violence as a primary outcome, and on bullying as a secondary outcome. It builds on the knowledge about the effectiveness of Dat-e Adolescence in comparison to previous assessments [37], examining the program’s effects at follow-up (6 months apart) and assessing its efficacy on other behavioral outcomes. This research seeks to contribute to the debate on the efficacy of dating violence programs and to test their effects on preventing other related forms of interpersonal violence, for example, bullying. We hypothesized that the Dat-e Adolescence program would yield substantial reductions in primary and secondary outcomes at follow-up. This hypothesis is based on previous research that addressed the fact that prevention programs failed to find positive reduction of dating aggression and victimization at post-test despite some effects have been found at medium term. In this respect, De la Rue et al. [48] concluded that the impact of these programs on changing aggressive behavior would require not only changing attitudes and knowledge of violence, but the development of adolescents’ socio-emotional skills that promote behavioral change when facing conflicts and disagreements with their partners. This skill-building training could help students to move to more healthy relationships, and later in time, to more positive and non-aggressive dating relationships. Since the program showed significant impact on improving emotional competence skills, such as students’ emotion regulation, and changing attitudes towards violence at post-test, we might expect some kind of delayed effects on dating violence at medium-term. Moreover, considering that poor anger regulation [25] and low self-esteem [49,50,51] are risk factors involved in other behavioral problems, such as bullying, we also expect the program to have an effect on this form of interpersonal violence. 

## 2. Materials and Methods

### 2.1. Participants

A total of 2050 adolescents participated in the study. For the main purpose of the study, they were selected based on having had romantic experience (*n* = 1423); 51.8% were male (*n* = 734), with ages ranging from 11 to 19 years (*M* = 14.98; SD = 1.39). 48.6% were in the first two-year cycle of high-school education (*n* = 691) and 51.4% in the second two-year cycle (*n* = 732). 830 participants studied in the province of Seville (58.3%) while 593 studied in the province of Córdoba (41.7%). Regarding romantic experience at Wave 1, 684 students had been in a relationship more than two months ago (48.1%); 305 had dated somebody in the last two months (21.4%); and 434 participants were in a current relationship (30.5%). 95.8% identified themselves as heterosexual or straight (*n* = 1357); 1% as gay or lesbian (*n* = 14); 1.6% as bisexual (*n* = 23); 0.2% as pansexual or demisexual (*n* = 3); and 1.3% did not know (*n* = 19). Around 95% of participants were born in Spain (*n* = 1357). The experimental group comprised 557 adolescents (53.8% male; age *M* = 14.88; SD = 1.30) from four high schools. The control group comprised 866 students (50.5% male; age *M* = 15.04; SD = 1.49) from three high schools. No differences in gender, X^2^(1) = 1.486; *p* = 0.231; CC = 0.03, or age, *F*(1, 1415) = 3.121; *p* = 0.077; eta^2^ = 0.002, were found between the control and experimental groups.

### 2.2. Attrition Analysis 

**Figure 1:**
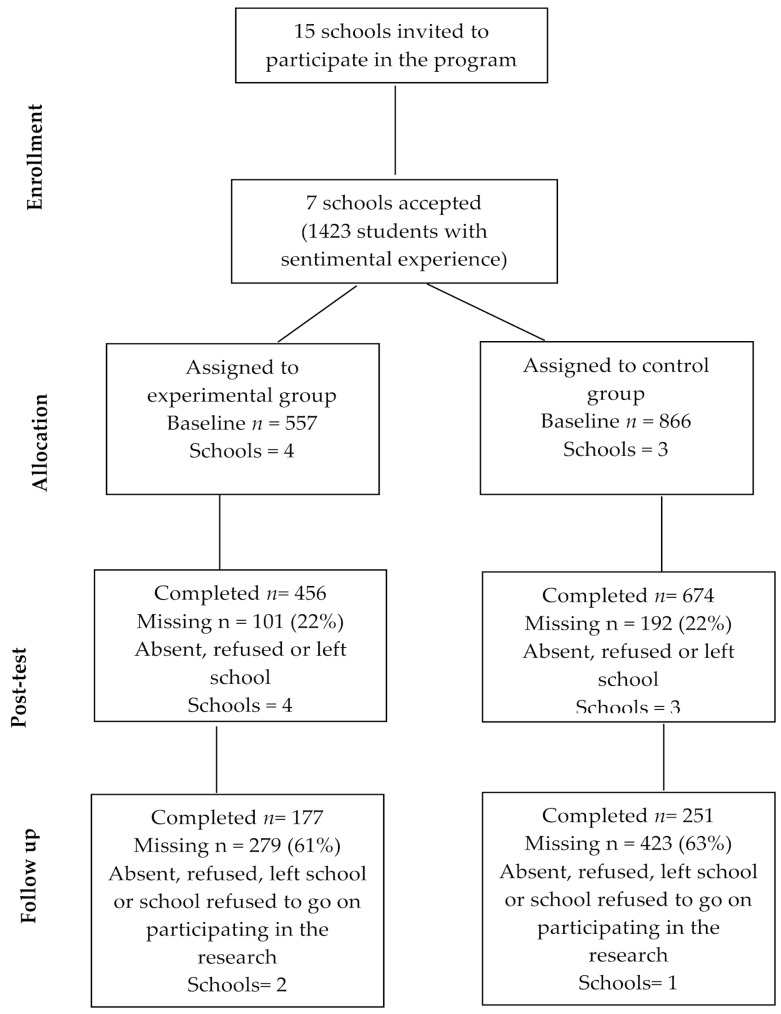
Flowchart of the recruitment and retention of participants in the evaluation.

Regarding attrition, 17% of participants completed the pre-test measure only (*n* = 242); 52.9% completed the pre-test and post-test measures (*n* = 753); and 30.1% of participants completed the pre-test, post-test, and follow-up measures (*n* = 428). Similar attrition rates were found for both groups, *X^2^*(2) = 1.932; *p* = 0.381. Specifically, 17.9% vs. 15.6% of control and experimental group participants, respectively, completed the pre-test measure; 53.1% vs. 52.6% of control and experimental group participants, respectively, completed the pre-test and post-test measures; and 29% vs. 31.8% of control and experimental group participants, respectively, completed all three waves. The high attrition rates observed at follow-up were due to four out of seven schools (experimental group = two schools, control group = two schools) choosing not to participate at follow-up (see flowchart, Figure 1). To establish similarities and differences between schools with follow-up and schools without follow-up, we compared the outcomes and sociodemographic variables at baseline level for both groups. No baseline differences for outcomes and sociodemographic variables were found between schools that participated at post-test and schools that participated at follow-up (see Table 1). For this reason, we decided to analyze intervention efficacy across all seven schools. 

We explored pre-test differences in outcomes and sociodemographic variables among participants with different attrition characteristics (pre-test only, pre-test and post-test, and all three waves; see Table 1). No gender differences were found between attrition groups, X^2^(2) = 3.342; *p* = 0.188; CC = 0.05, nor for control group attrition, X^2^(2) = 4.103; *p* = 0.129; CC = 0.07, or for experimental group attrition, X^2^(2) = 4.979; *p* = 0.083; CC = 0.09. Univariate general linear models (GLM) were estimated to analyze the association between age, baseline outcomes, treatment condition, and attrition type. Baseline outcomes and age were identified as the dependent variables, whereas treatment condition and attrition type were the fixed factors. Differences were observed in age, *F*(2, 1415) =12.006; *p* = 0.000; eta^2^ = 0.017, moderate physical aggression, *F*(2, 1368) = 7.355; *p* = 0.001; eta^2^ = 0.011, moderate physical victimization, *F*(2, 1374) = 4.690; *p* =0.009; eta^2^ = 0.007, severe physical aggression, *F*(2, 1366) = 6.550; *p* = 0.001; eta^2^ = 0.010, bullying aggression, *F*(2, 1396) = 5.891; *p* = 0.003; eta^2^ = 0.008, and bullying victimization, *F*(2, 1402) = 4.867; *p* = 0.008; eta^2^ = 0.007, between pre-test measure only, post-test, and follow-up participants.

Specifically, students who only participated at the pre-test were older and more involved in moderate physical aggression, moderate physical victimization, severe physical aggression, and bullying aggression compared to their three-wave counterparts. However, this trend was not observed for bullying victimization; pre-test-only participants were less involved than their three-wave peers. No differences were found in severe physical victimization, *F*(2, 1373) = 2.058; *p* = 0.128; eta^2^ = 0.003, sexual aggression, *F*(2, 1361) = 1.037; *p* = 0.355; eta^2^ = 0.002, or sexual victimization, *F*(2, 1370) = 0.240; *p* = 0.787; eta^2^ = 0.000, among participants with different attrition characteristics. Finally, no differences were found in moderate physical aggression, *F* (2, 1368) = 0.259; *p* = 0.772; eta^2^ = 0.000, moderate physical victimization, *F* (2, 1374) = 0.653; *p* = 0.521; eta^2^ = 0.001, severe physical aggression, *F*(2, 1366) = 2.005; *p* = 0.135; eta^2^ = 0.003, severe physical victimization, *F*(2, 1373) = 0.612; *p* = 0.542; eta^2^ = 0.001, sexual aggression, *F*(2, 1361) = 0.113; *p* = 0.893; eta^2^ = 0.000, sexual victimization, *F*(2, 1370) = 0.876; *p* = 0.417; eta^2^ = 0.001, bullying aggression, *F*(2, 1396) = 0.964; *p* = 0.382; eta^2^ = 0.001, or bullying victimization, *F*(2, 1402) = 0.271; *p* = 0.763; eta^2^ = 0.000, for the interaction effect between treatment condition and attrition type, suggesting that attrition differences were similar for both control and experimental groups. Thus, we can conclude that the intervention efficacy results obtained are not influenced by sample attrition. 

### 2.3. Procedure 

A RCT design was used with a control group and an experimental group. Fifteen state high schools from the cities of Seville and Córdoba (Andalusia region) were randomly selected by the Educational Authority using a simple randomization procedure (a list of random numbers was generated following a computer-based program). Those numbers that coincided with the school identification numbers were picked for the study. Following approval from the Research Ethics Committee of the Autonomous Region of Andalusia (code: 0575-N-14), initial contact was made with all 15 schools in December 2015. All schools presented a medium economic, social and cultural level (ISC Index in Spain) in accordance with the ranking established by the autonomous region’s education authority. Meetings were held with the schools’ directors and counseling teams to inform them about the research, its objectives, its content and the procedure to be selected for the experimental or control groups. Seven out of 15 schools agreed to take part in the study. Informed consent forms were signed and the program details were forwarded to the families and school boards, the latter granting all centers permission to take part. The seven centers were randomly assigned to either an experimental or control group using a coin toss procedure. A member of the research group who was not in direct contact with the school did this work. The waiting list procedure was applied to the control schools that expressed interest in receiving intervention in future editions. Pre-test was carried out in January 2016, post-test in June 2016, and follow-up in December 2016. The intervention took place from February through May 2016 once a week during school hours. Anonymous self-report, paper-and-pencil questionnaires were administered in the three waves. In order to identify students at the three points in time, the names of the participants were linked to a code that was used in each wave. The code was created taking into account the school, the class, and the number of each student in his/her class (the class list having been sorted alphabetically). Data were collected during school hours. Students received no rewards or incentives for taking part.

### 2.4. Measures

#### 2.4.1. Sociodemographic Variables

An ad hoc questionnaire was devised to ask participants about their gender, age, sexual orientation, locality, and nationality.

#### 2.4.2. Dating Relationship Status

Two items from the *Dating Questionnaire* [52] were used to analyze relationship status. The first item, a multiple-choice question, assessed the participants’ romantic experience. The response options were as follows: (a) Yes, I’m currently dating someone; (b) I’m not currently dating anyone, but I have done in the last two months; (c) I’m not dating anyone right now but I was, more than two months ago; and (d) I’ve never dated anyone before. The second item asked about the length of the current or past relationship expressed in weeks. 

#### 2.4.3. Physical Violence

Moderate and severe physical violence was evaluated using an adapted version of the physical violence scale [53] from the *Conflict Tactics Scale* (CTS2) [54]. Nine items, measured on a 5-point Likert scale (0 = Never; 1 = rarely; 2 = sometimes; 3 = often; 4 = Always), assessed the frequency with which the adolescents, in a current or past relationship, had perpetrated or received physically violent behaviors in last six months (e.g., “to shove or throw against a wall”). According to the Spanish validation of the scale for adolescents [55], the first four items represent moderate physical acts and the last five items represent more severe physical acts. CFA for two correlated factors showed a good fit for aggression, X^2^(26) = 44.191; RMSEA = 0.023; CFI = 0.902, and for victimization, X^2^(24) = 51.182; RMSEA = 0.029; CFI = 0.907.

#### 2.4.4. Sexual Violence

Sexual violence was assessed using an adapted version of the sexual dating violence measure proposed by Foshee et al. [56]. Four items, measured on a 5-point Likert scale (0 = Never; 1 = rarely; 2 = sometimes; 3 = often; 4 = Always), assessed the frequency with which the adolescents, in a current or past relationship, had perpetrated or received sexually violent behaviors in last six months (‘‘to pressure or force the other to have sex’’; “to pressure or force the other to engage in a sexual act when they did not want to”; “to touch the other when they did not want to be touched”; “to make humiliating comments about sexual behavior”). CFA showed a good fit for aggression, X^2^(2) = 1.611; RMSEA = 0.000; CFI = 1.000, and for victimization, X^2^(2) = 0.725; RMSEA = 0.000; CFI = 1.000.

#### 2.4.5. Bullying 

Bullying perpetration and victimization was assessed using the Spanish version of the European Bullying Intervention Project Questionnaire (EBIP-Q) [57]. Fourteen items, measured on a 5-point Likert scale (0 = Never; 1 = once or twice; 2 = once or twice a month; 3 = once a week; 4 = More than once a week), assessed the frequency with which adolescents perpetrated and received direct physical abuse, indirect abuse, verbal abuse, psychological abuse, and social exclusion in the last two months (e.g., ”someone has spread rumors about me”). CFA showed a good fit for aggression, X^2^(12) = 77.772; RMSEA = 0.063; CFI = 0.934, and for victimization, X^2^(13) = 92.807; RMSEA = 0.066; CFI = 0.949.

### 2.5. Analysis Plan 

All analyses were performed using MPLUS 7 (Muthen & Muthen, Los Angeles, CA, USA) and SPSS 24 (SPSS Inc., Chicago, IL, USA). In order to test the comparability of the control and experimental groups, we analyzed the pre-test differences [58]. Considering the size of the sample, we included eta^2^ to estimate the effect size. 

Following Cheong et al.’s [59] guidelines, multiple-group latent growth models were performed to analyze the effect of intervention on dating violence and bullying. According to the authors, when a prevention program is effective, the treatment and control groups are expected to show different outcome growth. To test this hypothesis, we first analyzed (Step 1) whether the linear trajectory shape fits the data and examined whether growth rates differed by condition. To this end, the trajectory of the outcome was examined separately using a two-group model [59]. The factor loadings of the latent factors were specified to be equal across both groups. The factor loadings on the latent intercepts factor were fixed to 1.0 to define the initial starting point. The factor loadings for the slope were set to 0, 1, and 2 given the equal timing across waves. The residual variances of each wave measure were also equated across groups. The covariance between intercept and slope factor was freely estimated and allowed for differences across both groups. Next (Step 2), the common intercept was estimated and the control group’s slope factor mean was set to 0. In consequence, the slope mean of the treatment group captured the mean difference in the slope factor mean between both groups. The model fit was assessed to analyze the appropriateness of using current specification for both groups and to justify the use of program group membership as a causal variable for the different trajectory shapes. Additionally, the chi-square difference test was used to choose the best model. The maximum likelihood robust (MLR) estimation method was used to estimate the models, given that the variables presented normality problems. To avoid bias due to sample attrition, all models were estimated using the full information maximum likelihood (FIML) method. Effect size was computed adhering to Raudenbush and Liu’s [60] formula for GMA analyses, where between-group differences in mean growth rates is the numerator and the slope SD is the denominator of the coefficient [61]. The following indices were used to evaluate the model fit: the chi-square (X^2^) statistic; the root mean square error of approximation (RMSEA); and the comparative fit index (CFI), with cut-off points of 0.08 for RMSEA and 0.90 for CFI. SPSS 24 statistics software was used for the descriptive analyses and for attrition analysis.

## 3. Results

In order to test the comparability of the experimental and control groups, we analyzed the pretest differences [58] (see Table 2). 

No differences were found in moderate physical dating aggression, *F*(1, 1368) = 0.030; *p* = 0.863; eta^2^ = 0.000, moderate physical dating victimization, *F*(1, 1374) = 0.462; *p* = 0.497; eta^2^ = 0.000, severe physical dating aggression, *F*(1, 1366) = 3.455; *p* = 0.063; eta^2^ = 0.003, severe physical dating victimization, *F*(1, 1373) = 1.880; *p* = 0.171; eta^2^ = 0.001, sexual dating aggression, *F*(1, 1361) = 0.009; *p* = 0.923; eta^2^ = 0.000, sexual dating victimization, *F*(1, 1370) = 0.076; *p*= 0.783; eta^2^ = 0.000, bullying aggression, *F*(1, 1396) = 0.046; *p* = 0.830; eta^2^ = 0.000, and bullying victimization, *F*(1, 1402) = 1.207; *p* = 0.272; eta^2^ = 0.001, between the control and experimental groups at pre-test. The correlations among outcome variables are shown in Table 3. Dating violence and bullying were positively correlated. For each form of violence, measures of aggression and victimization showed a strong correlation. The effect size was large for the relationship between moderate physical dating aggression and victimization, for severe physical dating aggression and victimization, and for sexual dating aggression and victimization. The effect size was medium for the relationship between bullying aggression and victimization. 

### 3.1. Effect of the Dat-e Adolescence Program on Moderate Physical Dating Violence

For moderate physical dating aggression, the overall fit of the two-group model was unacceptable in terms of the CFI value, X^2^(7) = 12.980; RMSEA = 0.035; CFI = 0.652. Modification indices were analyzed, suggesting that the residual variance of the post-test measure was different across groups (MI = 8.790). When freely estimating the residual variance at post-test across groups, the model showed a good fit, X^2^(6) = 4.499; RMSEA = 0.000; CFI = 1.000. In Step 1, the growth rate factor mean for both groups was found to be negative and significant (See Table 4). This suggests that growth was not due to intervention (i.e., normative growth). 

For moderate physical dating victimization, the overall fit of the two-group model was acceptable, X^2^(7) = 3.487; RMSEA = 0.000; CFI = 1.000. In Step 1, we observed a significant decrease over time for the experimental group and a declining trend for the control group (See Table 4). This suggests that growth was not due to intervention (i.e., normative growth). 

### 3.2. Effect of the Dat-e Adolescence Program on Severe Physical Dating Violence

For severe physical dating aggression, the overall fit of the two-groups model was acceptable, X^2^(7) = 3.870; RMSEA = 0.000; CFI = 1.000. In Step 1, the mean and variance of the growth rate factors for the control group were not significant, whereas the slope mean for the experimental group was negative and significant (see Table 4). In light of this, the means and variances of the growth factors were constrained to be 0 for the control group (Step 2). This model showed a good fit, X^2^(10) = 5.120; RMSEA = 0.000; CFI = 1.000, and the chi-square difference test was not significant, Trd = 0.063; df = 3; *p* = 0.996, suggesting that the best model was the constrained model. In this model, the growth rate factor mean estimated for the treatment group was the estimated program effect. When comparing the average growth rates between both groups, the average growth rate for the treatment group was negative and statistically significant. The effect size was between small to medium (*d* = 0.25). These results indicate that the treatment group’s growth trajectory of severe physical aggression decreased significantly compared to the control group’s growth trajectory. For severe physical dating victimization, the overall fit of the two-groups model was acceptable, X^2^(8) = 7.900; RMSEA = 0.000; CFI = 1.000, with the residual variance of the follow-up measure fixed to 0 for both groups. In Step 1, the mean and variance of the growth rate factors for the control group were not significant, whereas the slope mean for the experimental group was negative and significant (see Table 4). In light of this, the means and variances of the growth factors were constrained to be 0 for the control group (Step 2). This model showed a good fit, X^2^(11) = 7.092; RMSEA = 0.000; CFI = 1.000, and the chi-square difference test was not significant, Trd = 0.815; df = 3; *p* = 0.846, suggesting that the best model was the constrained model. In this model, the growth rate factor mean estimated for the treatment group was the estimated program effect. When comparing the average growth rates between both groups, the average growth rate for the treatment group was negative and statistically significant. The effect size was between small to medium (*d* = 0.21). These results indicate that the treatment group’s growth trajectory of severe physical aggression decreased significantly compared to the control group’s growth trajectory. 

### 3.3. Effect of the Dat-e Adolescence Program on Sexual Dating Violence

For sexual dating aggression, the overall fit of the two-groups model was acceptable, X^2^(7) = 8.095; RMSEA = 0.015; CFI = 0.918, with the residual variance of the follow-up measure fixed to 0. In Step 1, the mean and variance of the growth rate factors for the control group were not significant, whereas the slope mean for the experimental group was negative and significant (see Table 4). In light of this, the means and variances of the growth factors were constrained to be 0 for the control group (Step 2). This model showed a good fit, X^2^(10) = 9.890; RMSEA = 0.000; CFI = 1.000, and the chi-square difference test was not significant, Trd = 0.268; df = 3; *p* = 0.966, suggesting that the best model was the constrained model. In this model, the growth rate factor mean estimated for the treatment group was the estimated program effect. When comparing the average growth rates between both groups, the average growth rate for the treatment group was negative and statistically significant. The effect size was between small to medium (*d* = 0.38). These results indicate that the treatment group’s growth trajectory of severe physical aggression decreased significantly compared to the control group’s growth trajectory. 

For sexual dating victimization, the overall fit of the two-groups model was not acceptable in terms of the CFI value, X^2^(7) = 15.290; RMSEA = 0.041; CFI = 0.808. Modification indices were analyzed, suggesting that the residual variance of the post-test measure was different across groups (MI = 11.383). When freely estimating the residual variance at post-test across groups, the model showed a good fit, X^2^(6) = 7.740; RMSEA = 0.020; CFI = 0.960. In Step 1, the mean and variance of the growth rate factors for the control group were not significant, whereas the slope mean for the experimental group was negative and significant (see Table 4). In light of this, the means and variances of the growth factors were constrained to be 0 for the control group (Step 2). This model showed a good fit, X^2^(9) = 8.317; RMSEA = 0.000; CFI = 1.000, and the chi-square difference test was not significant, Trd = 0.351; df = 3; *p* = 0.950, suggesting that the best model was the constrained model. In this model, the growth rate factor mean estimated for the treatment group was the estimated program effect. When comparing the average growth rates between both groups, the average growth rate for the treatment group was negative and statistically significant. The effect size was between small to medium (*d* = 0.24). These results indicate that the treatment group’s growth trajectory of sexual victimization decreased significantly compared to the control group’s growth trajectory. 

### 3.4. Effect of the Dat-e Adolescence Program on Bullying 

For bullying aggression, the overall fit of the two-groups model was acceptable, X^2^(7) = 4.757; RMSEA = 0.000; CFI = 1.000. In Step 1, we observed a declining trend over time for the control group and non-significant changes for the experimental group (See Table 4). This result suggests that intervention did not modify bullying aggression. 

For bullying victimization, the overall fit of the two-groups model was acceptable, X^2^(7) = 8.321; RMSEA = 0.016; CFI = 0.997. In Step 1, the mean and variance of the growth rate factors for the control group were not significant, whereas the slope mean for the experimental group was negative and significant (see Table 4). In light of this, the means and variances of the growth factors were constrained to be 0 for the control group (Step 2). This model showed a good fit, X^2^(10) = 9.520; RMSEA = 0.000; CFI = 1.000, and the chi-square difference test was not significant, Trd = 1.478; df = 3; *p* = 0.687, suggesting that the best model was the constrained model. In this model, the growth rate factor mean estimated for the treatment group was the estimated program effect. When comparing the average growth rates between both groups, the average growth rate for the treatment group was negative and statistically significant. The effect size was large (*d* = 0.98). These results indicate that the treatment group’s growth trajectory of bullying victimization decreased significantly compared to the control group’s growth trajectory. 

## 4. Discussion

The present study is the first efficacy evaluation of the Dat-e Adolescence program to include a follow-up measurement. On the whole, the results lend clear support to the program’s effectiveness. In particular, students participating in the Dat-e Adolescence program, exhibited lower levels of severe physical dating violence (aggression and victimization); sexual dating violence (aggression and victimization); and bullying victimization six months after the intervention. 

Considering the magnitude of the intervention effects, we calculated Cohen’s *d* for the multiple-group latent growth models. The results revealed significant (small to medium) victimization and perpetration effects for this program at follow-up. Specifically, 0.21 for severe physical dating victimization; 0.25 for severe physical dating aggression; 0.24 for sexual dating victimization; 0.38 for sexual dating aggression; and 0.98 for bullying victimization. The results of this study are highly relevant in light of the fact that systematic reviews and meta-analyses [14,15,16,17] have emphasized how programs fail significantly to affect dating violence outcomes. Importantly, these effects at follow-up were not observed at the program’s post-test evaluation stage [37]. This difference between post-test and follow-up intervention efficacy warrants deeper discussion and hypotheses. In general terms, behaviors are resistant to change because they are consolidated in relational dynamics [62]. De la Rue et al. [14] suggest that behavior modification requires time and the development of socio-emotional skills that helps students to face with conflicts and problems within the couple in a healthy way. Applied to our program, because of the post-test evaluations took place immediately after program completion, it was difficult to observe any behavioral change. In this respect we can consider some mediating variables responsible for the delayed effects observed in this study. According to De la Rue et al. [48], programs need to include skill-building components to promote students’ behavioral changes when facing conflicts and problem within their romantic relationships. These socio-emotional skills would lead to healthier and more positive relationships that could prevent the expression of aggressive behaviors in dating couples. In the first efficacy assessment of the Dat-e Adolescence program, we found that students of the treatment conditions not only modified their beliefs about love and couple violence, but they also improved their socio-emotional skills. Specifically, after the intervention students showed a better control of anger, reduced the duration of anger episodes and the frequency with which they engaged in fights and arguments. Moreover, participants also reduced their negative feelings toward themselves, improving their self-esteem. We could hypothesize that the change observed in dating aggressive behavior at follow-up can be explained by means of the improved emotion regulations skills at post-test and the change of normative beliefs about love and violence shared by the peer group. Future mediation analysis could test this hypothesis confirming if the change in the behavior is produced as a result of these socio-emotional skills and the change in personal and peers’ attitudes towards violence. 

At follow-up, intervention and control-group students no longer showed significant differences in moderate physical dating aggression and victimization. Mean growth rates were negative in both groups, which potentially points to normative growth. To our knowledge, only one program has assessed its efficacy by differentiating between moderate and severe forms of physical dating violence [11,20]. It was Safe Dates, which successfully reduced moderate and severe physical aggression as well as moderate victimization, suggesting that moderate forms are more susceptible to change post-intervention. Unlike Safe Dates, our study reported clearer and stronger changes in more severe forms of dating violence (severe physical violence and sexual violence) than in moderate physical forms. It should be noted that decreased levels of aggression and victimization of moderate physical forms were observed in the experimental group. However, moderate physical victimization and aggression also decreased in the control group. This precludes obtaining conclusive results regarding program efficacy in relation to moderate physical violence. Given few programs have analyzed their effectiveness by comparing its impact on moderate and severe forms of physical violence, and taking into account that our results are inconclusive, future studies deepening this aspect are needed.

Considering the intervention’s significant effect on bullying victimization, we can confirm that the results are also very meaningful. We hypothesized that the Dat-e Adolescence program would lead to a reduction in different problem behaviors because bullying and dating violence share protective and risk factors over which the program intervenes [9,25,63] such as emotional competence. From a developmental perspective, bullying has been described as a stepping-stone of dating violence given that relational dynamics are transferred from one context to another [9]. As such, it is possible to see adolescents modifying their behavior not only with their partner but also with their peers after receiving the program. However, the program did not modify bullying aggression. This may in part be due to the program content itself. In fact, the general skills and strategies trained in the program, such as communication, emotion regulation and coping strategies, provide victims of bullying with the necessary resources. However, we could hypothesize that this more general content, geared toward positive development, is not enough to modify aggressive behavior among peers. Dat-e Adolescence was designed originally to be a dating violence prevention program, and therefore some of the contents, including beliefs and attitudes toward violence, are specific to couples and not to peer violence. Taken together, and considering the promising effects on peer victimization, future programs should contextualize dating violence programs on a more general framework of interpersonal violence in adolescence, including new contents more closely related to peer aggression, in order to maximize the impact of these programs on different aggressive behaviors [13]. 

In general, the findings are considerably significant both for dating violence prevention policies in Spain and for interpersonal violence in adolescent research. The Dat-e Adolescence program was born out of a context in which most programs evaluated and analyzed under the umbrella of systematic reviews and meta-analyses come from the United States and Canada [14,15]. However, in Europe, the efficacy of already implemented programs remains an unknown [2]. To date, no previous Spanish prevention programs have been assessed using a RCT design and a large sample. Furthermore, none have delivered promising results in dating violence prevention, and to our knowledge, there is no evidence of a single program that prevents different behavioral problems in adolescents in Spain. Yet in our study, an experimental design, a large sample, and psychometrically sound measures were used. Data were also analyzed using rigorous statistical methods, which yielded promising results. 

Despite the positive results of the Dat-e Adolescence program’s effectiveness, future studies should focus on some key questions. A second independent trial is needed to confirm the positive results described in this study and in the program’s post-test evaluation [37], adhering to standards of evidence [57,64]. In addition, research should look into whether these positive results can be replicated by changing an important program component, namely the trainers. In this first edition, the first five sessions were led by researchers; however, we believe that teachers and/or psychologists can be equally good trainers. Teacher-led training offers the opportunity to test the program’s efficacy under more real and natural conditions. The Dat-e Adolescence program comes with a detailed manual, containing easy-to-follow and specific descriptions about the aims of each session, standardized instructions, and materials that can be used for this purpose. Furthermore, much more needs to be done to address several key issues concerning the effectiveness of this type of intervention; for example, the mechanism of change. In this line, the design of the Dat-e Adolescence program was based mainly on two models; DDS model [44] and a peer-led training. A systematic review of the efficacy of dating violence prevention [17] has emphasized the difficulty of preventing this complex problem where multiple risk factors intersect. For this reason, O’Leary and Slep [17] proposed to consider the DDS model [44] in the formulation of prevention programs and to include their ecological approach in the intervention design. In this line, it is necessary to analyze the impact that protective and risk factors may have on the problem using mediational models. This can help us to understand the mechanisms and processes that lead to change [2] and to test the conceptual models developed to better explain interpersonal violence in adolescence [44,64]. Moreover, the Dat-e Adolescence program included peer-led training in line with previous dating violence and bullying prevention programs [24,31]. To determine the benefits of a peer-led model component requires testing the efficacy of the program with this component and without it, and then to compare the results. In sum, it is necessary to improve our knowledge about what ingredients are responsible for the positive results, in terms of a cost-benefit approach [65].

Despite its strengths, the study has several limitations. Self-report measures were used which can be affected by social desirability bias. Our results are only generalizable to schools with similar characteristics. This study involved schools with a medium socioeconomic level, meaning it was not representative of very low-risk or very high-risk schools. In addition, a sizeable proportion of incomplete data was observed at follow-up, as four schools decided not to participate at this wave. Attrition rates were higher in older students, so future studies could benefits by including age as covariate. It is true that Attrition analysis revealed that the results were not influenced by attrition but the inclusion of this covariate could help us to conclude about the effect of age more accurately. 

Closely related to this already mentioned limitation is the fact that that we did not include the differential effect of the program on boys and girls. In this respect, as reported in previous systematic reviews, an important number of dating violence prevention programs have shown to be more effective to reduce dating aggression in boys than in girls [48], which could be understood as an indicator that the programs contribute to stop the circle of violence of boys towards girls. Future studies should consider the effect of gender in the results in order to confirm the effect of this program in the reduction of gender violence. 

## 5. Conclusions

Romantic relationships are a veritable challenge for adolescents. When faced with this scenario; some adolescents will acquire the strategies and skills enabling them to establish healthy relationships with their partners. According to the Center for Disease Control and Prevention [66], romantic relationships are healthy when the following criteria are met; both partners share the conviction that conflicts can be resolved in a non-violent way; they have the ability to adapt to and negotiate stressful situations, making decisions together; communication skills and/or assertive communication are present; couples accept that their respective partners have the right to practice their autonomy; and mutual trust lies at the heart of the relationship. Dating violence prevention programs can be a valuable resource to help young people face this difficult task and encourage them to build respectful relationships, thus encouraging positive development for both members of the dyad. In the present study, the results corresponding to the program’s efficacy suggest that Dat-e Adolescence can mitigate severe forms of physical dating violence and sexual dating violence as well as bullying victimization. This last finding is critical given that cross-cutting interventions are deemed more efficient in terms of time and resources.

## Figures and Tables

**Table 1:** Attrition analysis. Comparison effects of attrition at school level and at participant level.

Outcomes		Comparison Effect of Attrition for Schools			Comparison Effect of Treatment Condition and Attrition for Participants								
		Schools at Post-Test (7 Schools)	Schools at Follow-Up (3 Schools)	Diff.	Participants with Only Pre-Test			Participants with Pre-Test and Post-Test			Participants with Three Waves		
					CG	EG	Total	CG	EG	Total	CG	EG	Total
Moderate Physical dating aggression		0.063 (0.235)	0.057 (0.214)	*T*(1366) = −10.027; *p* = 0.304; *d* = −0.06	0.096 (0.309)	0.097 (0.287)	0.096 (0.301)	0.069 (0.232)	0.056 (0.244)	0.064 (0.236)	0.023 (0.137)	0.027 (0.141)	0.025 (0.139)
Moderate Physical dating victimization		0.075 (0.289)	0.068 (0.288)	*T*(1372) = −0.391; *p* = 0.696; *d* = 0.02	0.097 (0.353)	0.125 (0.383)	0.107 (0.363)	0.085 (0.306)	0.072 (0.294)	0.080 (0.303)	0.029 (0.141)	0.051 (0.257)	0.038 (0.198)
Severe Physical dating aggression		0.043 (0.195)	0.047 (0.207)	*T*(1364) = 0.389; *p* = 0.697; *d* = 0.02	0.052 (0.186)	0.110 (0.327)	0.073 (0.246)	0.051 (0.221)	0.046 (0.227)	0.049 (0.223)	0.013 (0.076)	0.028 (0.130)	0.019 (0.103)
Severe Physical dating victimization		0.044 (0.185)	0.050 (0.237)	*T*(1371) = 0.577; *p* = 0.564; *d* = 0.03	0.050 (0.164)	0.073 (0.213)	0.058 (0.183)	0.053 (0.242)	0.054 (0.254)	0.053 (0.247)	0.017 (0.080)	0.045 (0.195)	0.029 (0.141)
Sexual dating aggression		0.044 (0.251)	0.034 (0.156)	*T*(1359) = −0.908; *p* = 0.364; *d* = −0.05	0.037 (0.162)	0.028 (0.112)	0.034 (0.147)	0.045 (0.208)	0.052 (0.333)	0.048 (0.264)	0.028 (0.131)	0.034 (0.148)	0.030 (0.139)
Sexual dating victimization		0.065 (0.293)	0.077 (0.275)	*T*(1368) = 0.790; *p* = 0.430; *d* = 0.04	0.072 (0.338)	0.045 (0.130)	0.062 (0.282)	0.089 (0.329)	0.066 (0.269)	0.075 (0.307)	0.054 (0.205)	0.081 (0.290)	0.065 (0.245)
Bullying aggression		0.439 (0.608)	0.392 (0.492)	*T*(1394) = −10.563; *p* = 0.118; *d* = −0.08	0.426 (0.539)	0.466 (0.559)	0.440 (0.546)	0.439 (0.626)	0.477 (0.624)	0.454 (0.625)	0.369 (0.451)	0.314 (0.399)	0.346 (0.431)
Bullying victimization		0.681 (0.750)	0.687 (0.749)	*T*(1400) = −0.135; *p* = 0.893; *d* = −0.01	0.578 (0.726)	0.573 (0.631)	0.576 (0.693)	0.709 (0.768)	0.782 (0.797)	0.737 (0.779)	0.618 (0.649)	0.701 (0.805)	0.652 (719)
Gender	Boys	407 (500.7%)	327 (530.2%)	X^2^(1) = 0.862; *p* = 0.362; CC = 0.03	68 (440.7%)	54 (620.8%)	122 (510.3%)	229 (490.9%)	146 (490.8%)	375 (490.9%)	138 (55%)	99 (550.9%)	237 (550.4%)
	Girls	396 (490.3%)	288 (460.8%)		84 (550.3%)	32 (370.2%)	116 (480.7%)	230 (500.1%)	147 (500.2%)	377 (500.1%)	113 (45%)	78 (440.1%)	191 (440.6%)
Age		140.985 (10.426)	140.977 (10.353)	*T*(1413) = −0.103; *p* = 0.918; *d* = −0.00	150.362 (10.715)	150.128 (10.423)	150.278 (10.621)	150.112 (10.433)	140.929 (10.311)	150.041 (10.389)	140.724 (10.227)	140.695 (10.202)	140.712 (10.216)

Note: CG = control group; EG = experimental group; Mean and (Standard Deviation) are displayed in the table except for gender; *T* = Student’s T; *p* = *p*-value; *d* = Cohen’s *d*; X^2^ = Chi-squared; CC = Contingency coefficient.

**Table 2:** Descriptive statistics in the three waves for primary and secondary outcomes.

Outcomes	Group	Pre-Intervention			Post-Intervention			Follow-Up		
		M	SD	*n*	M	SD	*n*	M	SD	*n*
Moderate physical aggression	Control group	0.061	0.227	833	0.066	0.317	623	0.024	0.111	220
	Experimental group	0.053	0.224	535	0.036	0.189	421	0.021	0.094	154
Moderate physical victimization	Control group	0.071	0.280	835	0.075	0.322	629	0.039	0.161	220
	Experimental group	0.073	0.300	539	0.050	0.275	420	0.027	0.116	155
Severe physical aggression	Control group	0.040	0.184	831	0.037	0.222	623	0.023	0.094	220
	Experimental group	0.050	0.222	535	0.027	0.168	421	0.005	0.039	154
Severe physical victimization	Control group	0.041	0.194	835	0.044	0.229	629	0.027	0.119	230
	Experimental group	0.054	0.230	538	0.039	0.256	420	0.015	0.100	155
Sexual aggression	Control group	0.039	0.181	829	0.039	0.201	623	0.033	0.167	220
	Experimental group	0.042	0.259	532	0.035	0.181	420	0.015	0.082	154
Sexual victimization	Control group	0.071	0.299	833	0.060	0.259	629	0.058	0.239	220
	Experimental group	0.067	0.260	537	0.082	0.339	419	0.047	0.191	155
Bullying aggression	Control group	0.417	0.566	854	0.392	0.535	666	0.366	0.470	243
	Experimental group	0.423	0.555	542	0.408	0.588	450	0.319	0.425	173
Bullying victimization	Control group	0.659	0.729	857	0.649	0.737	667	0.626	0.674	243
	Experimental group	0.724	0.779	545	0.668	0.736	449	0.600	0.726	173

**Table 3:** Correlations among outcome variables.

Outcomes	Moderate Dating Physical Victimization	Severe Dating Physical Aggression	Severe Dating Physical Victimization	Sexual Dating Aggression	Sexual Dating Victimization	Bullying Aggression	Bullying Victimization
Moderate physical dating aggression	0.73 ***	0.48 ***	0.41 ***	0.30 ***	0.21 ***	0.20 ***	0.05
Moderate physical victimization	1	0.48 ***	0.53 ***	0.23 ***	0.29 ***	0.18 ***	0.14 ***
Severe physical aggression		1	0.70 ***	0.45 ***	0.27 ***	0.22 ***	0.10 ***
Severe physical victimization			1	0.37 ***	0.38 ***	0.16 ***	0.14 ***
Sexual aggression				1	0.49 ***	0.13 ***	0.05
Sexual victimization					1	0.06 *	0.18 ***
Bullying aggression						1	0.46 ***
Bullying victimization							1

Note: * *p* < 0.05; *** *p* < 0.001.

**Table 4:** Multiple-group estimated components of growth curves.

Outcome	Group	Step 1: Model Free				Step 2: Model Constrained			
		Intercept		Slope		Intercept		Slope	
		Mean	Variance	Mean	Variance	Mean	Variance	Mean	Variance
Moderate physical dating aggression	Control group	0.059 (0.006) ***	0.020 (0.009) **	−0.014 (0.006) *	0.004 (0.005)				
	Experimental group	0.059 (0.006) ***	0.020 (0.009) **	−0.019 (0.005) ***	0.007 (0.004)				
Moderate physical victimization	Control group	0.073 (0.008) ***	0.059 (0.034) ^†^	−0.015 (0.008) ^†^	0.020 (0.017)				
	Experimental group	0.073 (0.008) ***	0.059 (0.034) ^†^	−0.023 (0.006) ***	0.026 (0.009) ^†^				
Severe physical aggression	Control group	0.045 (0.005) ***	0.014 (0.008) ^†^	−0.005 (0.005)	0.000 (0.002)	0.042 (0.005) ***	0.015 (0.006) **	0	0
	Experimental group	0.045 (0.005) ***	0.014 (0.008) ^†^	−0.020 (0.003) ***	0.004 (0.002) ^†^	0.042 (0.005) ***	0.015 (0.006) **	−0.018 (0.003) ***	0.005 (0.002) *
Severe physical victimization	Control group	0.046 (0.007) ***	0.040 (0.038)	−0.012 (0.016)	0.019 (0.034)	0.046 (0.005) ***	0.020 (0.006) **	0	0
	Experimental group	0.046 (0.007) ***	0.040 (0.038)	−0.015 (0.005) **	0.009 (0.009)	0.046 (0.005) ***	0.020 (0.006) **	−0.015 (0.004) **	0.005 (0.003) ^†^
Sexual aggression	Control group	0.041 (0.006) ***	0.014 (0.008)	−0.001 (0.006)	0.001 (0.007)	0.041 (0.005) ***	0.015 (0.006) *	0	0
	Experimental group	0.041 (0.006) ***	0.014 (0.008)	−0.012 (0.004) **	0.001 (0.003)	0.041 (0.005) ***	0.015 (0.006) *	−0.012 (0.004) **	0.001 (0.003)
Sexual victimization	Control group	0.070 (0.007) ***	0.032 (0.022)	−0.005 (0.007)	0.002 (0.010)	0.068 (0.007) ***	0.034 (0.015) *	0	0
	Experimental group	0.070 (0.007) ***	0.032 (0.022)	−0.014 (0.006) *	0.003 (0.005)	0.068 (0.007) ***	0.034 (0.015) *	−0.013 (0.005) *	0.003 (0.004)
Bullying aggression	Control group	0.421 (0.015) ***	0.201 (0.033) ***	−0.022 (0.013) ^†^	0.025 (0.017)				
	Experimental group	0.421 (0.015) ***	0.201 (0.033) ***	−0.024 (0.017)	0.022 (0.018)				
Bullying victimization	Control group	0.684 (0.020) ***	0.320 (0.043) ***	−0.020 (0.018)	0.008 (0.026)	0.676 (0.018) ***	0.301 (0.030) ***	0	0
	Experimental group	0.684 (0.020) ***	0.320 (0.043) ***	−0.047 (0.020) *	0.009 (0.027)	0.676 (0.018) ***	0.301 (0.030) ***	−0.044 (0.020) *	0.002 (0.019)

Note: In parenthesis SE is reported. ^†^ *p* < 0.10; * *p* < 0.05; ** *p* < 0.01; *** *p* < 0.001; In Step 1 the means of the growth rate were freely estimated in both groups; In Step 2, the mean of the growth rate was constrained to 0 for the control group and freely estimated in the experimental group.

## References

[1] Centers for Disease Control and Prevention (2017). Preventing Intimate Partner Violence across the Lifespan: A Technical Package of Programs, Policies, and Practices.

[2] Leen E., Sorbring E., Mawer M., Holdsworth E., Helsing B., Bowen E. (2013). Prevalence, dynamic risk factors and the efficacy of primary interventions for adolescent dating violence: An international review. Aggress. Violent Behav..

[3] Rubio-Garay F., López-González M.A., Carrasco M.Á., Javier Amor P. (2017). Prevalencia de la Violencia en el Noviazgo: Una Revisión Sistemática. Papeles del Psicólogo.

[4] Wincentak K., Connolly J., Card N. (2017). Teen Dating Violence: A Meta-Analytic Review of Prevalence Rates. Psychol Violence.

[5] Stonard K.E., Bowen E., Lawrence T.R., Price S.A. (2014). The relevance of technology to the nature, prevalence and impact of Adolescent Dating Violence and Abuse: A research synthesis. Aggress. Violent Behav..

[6] Exner-Cortens D., Eckenrode J., Rothman E. (2013). Longitudinal associations between teen dating violence victimization and adverse health outcomes. Pediatrics.

[7] Cutbush S., Williams J., Miller S. (2016). Teen Dating Violence, Sexual Harassment, and Bullying Among Middle School Students: Examining Mediation and Moderated Mediation by Gender. Prev. Sci..

[8] Espelage D.L., Hong J.S., Valido A., Wolfe D.A., Temple J.R. (2019). Associations Among Family Violence, Bullying, Sexual Harassment, and Teen Dating Violence. Adolescent Dating Violence. Theory, Research and Prevention.

[9] Josephson W.L., Pepler D. (2012). Bullying: A stepping stone to dating aggression. Int. J. Adolesc. Med. Health.

[10] Pepler D.J., Craig W.M., Connolly J.A., Yuile A., McMaster L., Jiang D. (2006). A developmental perspective on bullying. Aggress. Behav..

[11] Foshee V.A., Bauman K.E., Ennett S.T., Linder G.F., Benefield T., Suchindran C. (2004). Assessing the long-term effects of the Safe Dates program and a booster in preventing and reducing adolescent dating violence victimization and perpetration. Am. J. Public Health.

[12] Wolfe D.A., Crooks C., Jaffe P., Chiodo D., Hughes R., Ellis W., Stitt L., Donner A. (2009). A school-based program to prevent adolescent dating violence: A cluster randomized trial. Arch. Pediatr. Adolesc. Med..

[13] Taylor B.G., Stein N.D., Mumford E.A. (2013). Shifting Boundaries: An Experimental Evaluation of a Dating Violence Prevention Program in Middle Schools. Prev. Sci..

[14] De La Rue L., Joshua P., Dorothy E., Pigott T. (2014). School-based interventions to reduce dating and sexual violence: A systematic review. Campbell Syst. Rev..

[15] Fellmeth G.L.T., Heffernan C., Nurse J., Habidula S., Sethi D. (2014). Educational and skills-based interventions for preventing relationship and dating violence in adolescents and young adults: A systematic review. Campbell Syst. Rev..

[16] Martínez Gómez J.A., Rey-Anacona C.A. (2014). Prevention of Dating Violence: A Review of Programs Published Between 1990 and 2012. Pensam Psicológico.

[17] O’Leary K.D., Slep A.M.S. (2012). Prevention of Partner Violence by Focusing on Behaviors of Both Young Males and Females. Prev. Sci..

[18] Foshee V.A., Reyes H.L.M., Ennett S.T., Cance J.D., Bauman K.E., Bowling M. (2012). Assessing the Effects of Families for Safe Dates, a Family-Based Teen Dating Abuse Prevention Program. J. Adolesc. Health.

[19] Wolfe D.A., Wekerle C., Scott K., Straatman A.L., Grasley C., Reitzel-Jaffe D. (2003). Dating violence prevention with at-risk youth: A controlled outcome evaluation. J. Consult. Clin. Psychol..

[20] Foshee V.A., Bauman K.E., Ennett S.T., Suchindra C., Benefield T., Linder G.F. (2005). Assessing the effects of the dating violence prevention program “safe dates” using random coefficient regression modeling. Prev. Sci..

[21] Coker A.L., Bush H.M., Cook-Craig P.G., DeGue S.A., Clear E.R., Bancato C.J., Fisher B.S., Recktenwald E.A. (2017). RCT Testing Bystander Effectiveness to Reduce Violence. Am. J. Prev. Med..

[22] Levesque D.A., Johnson J.L., Welch C.A., Prochaska J.M., Paiva A.L. (2016). Teen Dating Violence Prevention: Cluster-Randomized Trial of Teen Choices, an Online, Stage-Based Program for Healthy, Nonviolent Relationships. Psychol. Violence.

[23] Miller E., Tancredi D.J., Mccauley H.L., Decker M.R., Virata M.C.D., Anderson H.A., O’Connor B., Silverman J.G. (2013). One-Year Follow-Up of a Coach-Delivered Dating Violence Prevention Program. Am. J. Prev. Med..

[24] Connolly J., Josephson W., Schnoll J., Simkins-Strong E., Pepler D., MacPherson A., Wieser J., Moran M., Jiang D. (2015). Evaluation of a youth-led program for preventing bullying, sexual harassment, and dating aggression in middle schools. J. Early Adolesc..

[25] Foshee V.A., Reyes H.L.M., Chen M.S., Ennett S.T., Basile K.C., DeGue S., Vivolo-Kantor A.M., Moracco K.E., Bowling J.M. (2016). Shared Risk Factors for the Perpetration of Physical Dating Violence, Bullying, and Sexual Harassment Among Adolescents Exposed to Domestic Violence. J. Youth Adolesc..

[26] Foshee V.A., Reyes H.L.M., Whitaker D.J., Lutzker J.R. (2009). Primary prevention of adolescent dating abuse perpetration: When to begin, whom to target, and how to do it. Preventing Partner Violence: Research and Evidence-Based Intervention Strategies.

[27] Whitaker D.J., Murphy C.M., Eckhardt C.I., Hodges A.E., Cowart M. (2013). Effectiveness of Primary Prevention Efforts for Intimate Partner Violence. Partner Abuse.

[28] De Koker P.B., Mathews C.C., Zuch M.D., Bastien S., Mason-Jones A.J. (2014). systematic review of interventions for preventing adolescent intimate partner violence. J. Adolesc. Health.

[29] Storer H.L., Casey E., Herrenkohl T. (2016). Efficacy of Bystander Programs to Prevent Dating Abuse Among Youth and Young Adults: A Review of the Literature. Trauma Violence Abuse.

[30] Williford A., Elledge L.C., Boulton A.J., DePaolis K.J., Little T.D., Salmivalli C. (2013). Effects of the Kiva Antibullying Program on Cyberbullying and Cybervictimization Frequency Among Finnish Youth. J. Clin. Child Adolesc. Psychol..

[31] Palladino B.E., Nocentini A., Menesini E. (2016). Evidence-Based Intervention against Bullying and Cyberbullying: Evaluation of the NoTrap! Program in Two Independent Trials. Aggress. Behav..

[32] Del Rey R., Casas J.A., Ortega-Ruiz R. (2012). El programa ConRed: Una práctica basada en la evidencia. Comunicar.

[33] Hernando Á. (2007). La prevención de la violencia de género en adolescentes. Una experiencia en el ámbito educativo. Apunt Psicol..

[34] Garrido V., Casas M. (2009). La prevención de la violencia en la relación amorosa entre adolescentes a través del taller « La Máscara del Amor ». Rev. Educ..

[35] Fernández-González L., Muñoz-Rivas M.J. (2013). Evaluación De Un Programa De Prevención De La Violencia En Las Relaciones De Noviazgo: Indicaciones Tras Un Estudio Piloto. Behav. Psychol..

[36] Muñoz B., Ortega-Rivera F., Sánchez-Jiménez V. (2013). El DaViPoP: Un programa de prevención de violencia en el cortejo y las parejas adolescentes. Apunt Psicol..

[37] Sánchez-Jiménez V., Muñoz-Fernández N., Ortega-Rivera J. (2018). Efficacy evaluation of “Dat-e Adolescence”: A dating violence prevention program in Spain. PLoS ONE.

[38] Chiodo D., Crooks C.V., Wolfe D.A., McIsaac C., Hughes R., Jaffe P.G. (2012). Longitudinal Prediction and Concurrent Functioning of Adolescent Girls Demonstrating Various Profiles of Dating Violence and Victimization. Prev. Sci..

[39] Fernández-González L., Calvete E., Orue I. (2017). Adolescent Dating Violence Stability and Mutuality: A 4-Year Longitudinal Study. J. Interpers. Violence.

[40] Collins W.A., van Dulmen M., Crouter A.C., Booth A., Snyder A. (2016). “The course of the true love(s)…”: Origins and pathways in the development of romantic relationships. Romance and Sex in Adolescence and Emerging Adulthood: Risks and Opportunities.

[41] Espelage D.L., Holt M.K. (2007). Dating violence & sexual harassment across the bully-Victim Continuum among middle and high school students. J. Youth Adolesc..

[42] Sánchez-Jiménez V., Viejo C., Ortega-Ruíz R. (2012). El contexto de los iguales y de la pareja como factores predictores de la agresión física y sexual en las parejas adolescentes. Prolespsis.

[43] Reyes H.L.M., Foshee V.A., Bauer D.J., Ennett S.T. (2012). Heavy Alcohol Use and Dating Violence Perpetration During Adolescence: Family, Peer and Neighborhood Violence as Moderators. Prev. Sci..

[44] Capaldi D.M., Shortt J.W., Kim H.K., Pinsof W.M., Lebow J.L. (2005). A Life Span Developmental Systems Perspective on Aggression toward a Partner. Oxford Series in Clinical Psychology. Family Psychology: The Art of the Science.

[45] Capaldi D.M., Knoble N.B., Shortt J.W., Kim H.K. (2012). A Systematic Review of Risk Factors for Intimate Partner Violence. Partner Abuse.

[46] Vagi K.J., Rothman E., Latzman N.E., Tharp A.T., Hall D.M., Breiding M. (2014). Beyond Correlates: A Review of Risk and Protective Factors for Adolescent Dating Violence Perpetration. J. Youth Adolesc..

[47] Shortt J.W., Capaldi D.M., Kim H.K., Kerr D.C.R., Owen L.D., Feingold A. (2012). Stability of Intimate Partner Violence by Men across 12 Years in Young Adulthood: Effects of Relationship Transitions. Prev. Sci..

[48] De La Rue L., Polanin J.R., Espelage D.L., Pigott T.D. (2017). A Meta-Analysis of School-Based Interventions Aimed to Prevent or Reduce Violence in Teen Dating Relationships. Rev. Educ. Res..

[49] Fanti K.A., Henrich C. (2014). Effects of Self-Esteem and Narcissism on Bullying and Victimization during Early Adolescence. J. Early Adolesc..

[50] O’Moore M., Kirkham C. (2001). Self-Esteem and Its Relationship to Bullying Behaviour. Aggress. Behav..

[51] Patchin J.W., Hinduja S. (2016). Cyberbullying and self-esteem. J. Sch. Health.

[52] Connolly J., Craig W., Goldberg A., Pepler D. (2004). Mixed-Gender Groups, Dating, and Romantic Relationships in Early Adolescence. J. Res. Adolesc..

[53] Nocentini A., Menesini E., Pastorelli C., Connolly J., Pepler D., Craig W. (2011). Physical Dating Aggression in Adolescence. Cultural and Gender invariance. Eur. Psychol..

[54] Straus M. (1979). Measuring Intrafamily Conflict and Violence: The Conflict Tactics (CT) Scales. J. Marriage Fam..

[55] Viejo C., Sánchez-Jiménez V., Ortega-Ruiz R. (2014). Physical dating violence: The potential understating value of a bi-factorial model. Anales de Psicologia.

[56] Foshee V.A., Benefield T.S., Ennett S.T., Bauman K.E., Suchindran C. (2004). Longitudinal predictors of serious physical and sexual dating violence victimization during adolescence. Prev. Med..

[57] Ortega-Ruiz R., Del Rey R., Casas J.A. (2016). Evaluar el bullying y el cyberbullying validación española del EBIP-Q y del ECIP-Q. Psicol. Educ..

[58] Flay B., Biglan A., Boruch R., González F., Gottfredson D., Kellam S., Mościcki E.K., Schinke S., Valentine J.C., Ji P. (2005). Standards of Evidence Criteria for Efficacy, Effectiveness and Dissemination. Prev. Sci..

[59] Cheong J., MacKinnon D.P., Khoo S.L. (2003). Investigation of Mediational Processes Using Parallel Process Latent Growth Curve Modeling. Struct. Equ. Model..

[60] Raudenbush S.W., Xiao-Feng L. (2001). Effects of Study Duration, Frequency of Observation, and Sample Size on Power in Studies of Group Differences in Polynomial Change. Psychol. Methods.

[61] Feingold A. (2009). Trials in the Same Metric as for Classical Analysis. Psychol. Methods.

[62] Cornelius T.L., Resseguie N. (2007). Primary and secondary prevention programs for dating violence: A review of the literature. Aggress. Violent Behav..

[63] Espelage D.L., Low S.K., Anderson C., Ru L.D. (2014). Bullying, Sexual, and Dating Violence Trajectories from Early to Late Adolescence.

[64] Gottfredson D.C., Cook T.D., Gardner F.E.M., Gorman-Smith D., Howe G.W., Sandler I.N., Zafft K.M. (2015). Standards of Evidence for Efficacy, Effectiveness, and Scale-up Research in Prevention Science: Next Generation. Prev. Sci..

[65] Menesini E., Salmivalli C. (2017). Bullying in schools: The state of knowledge and effective interventions. Psychol. Health Med..

[66] Centers for Disease Control and Prevention (2008). Strategic Direction for Intimate Partner Violence Prevention: Promoting Respectful, Nonviolent Intimate Partner Relationships through Individual, Community, and Societal Change.

